# Histopathologic Characterization of Cutaneous Oxalosis Mimicking Calciphylaxis: Diagnostic Pitfalls and Polarized Light Evaluation

**DOI:** 10.1111/cup.70177

**Published:** 2026-07-13

**Authors:** Shrina Patel, Brad Graham, Kaci Reaves, Mya Phelps

**Affiliations:** ^1^ Department of Dermatology University of Texas at Tyler School of Medicine Tyler Texas USA; ^2^ U.S. Dermatology Partners Tyler Texas USA

## Abstract

Cutaneous oxalosis is an uncommon manifestation of primary hyperoxalosis, which can often resemble other cutaneous manifestations of end stage renal disease (ESRD), particularly calciphylaxis. We report the case of a 46 year old female with primary hyperoxaluria type one (PH1) believed to be well‐controlled on lumasiran, a small interfering ribonucleic acid therapy, which targets the mRNA responsible for producing hydroxyacid oxidase 1 (HAO1) and through reduction of this enzyme, decreases the hepatic overproduction of oxalate. The patient presented for dermatologic evaluation of presumed calciphylaxis. Repeated skin biopsies, however, confirmed cutaneous oxalosis despite perceived stable metabolic control with lumasiran. This case highlights the complexities and challenges in the diagnosis of cutaneous manifestations of chronic kidney disease and emphasizes the importance of histopathologic evaluation, even if the patient's primary hyperoxaluria is believed to be well‐controlled.

## 1 Introduction

Primary hyperoxalosis occurs due to inborn errors in metabolism of oxalate and glyoxylate [1]. With natural progression and chronicity, hyperoxalosis leads to renal deposition of calcium oxalate, causing nephrocalcinosis [2]. With progressive kidney injury, renal excretion of oxalate decreases and deposition expands to other organs resulting in systemic disease [3]. Cutaneous manifestations of oxalosis are rare and can mimic other manifestations of ESRD. In this report, we discuss a patient with primary hyperoxalosis, thought to be well‐controlled with lumasiran. This novel synthetic double‐stranded ribonucleic acid (RNA) interference molecule targets and effectively silences the mRNA blueprint for hydroxyacid oxidase 1 (HAO1), which in turn halts the production of the glycolate oxidase (GO) enzyme and thereby shuts down the upstream chemical pathway that leads to toxic oxalate accumulation [4]. The patient was referred by her nephrologist to dermatology for painful stellate skin ulcerations and livedo reticularis with urgent concern for calciphylaxis; however, repeated skin biopsies ultimately revealed cutaneous oxalosis.

## 2 Case Report

A 46‐year‐old female with a history of ESRD, primary hyperoxaluria type 1 (PH1), multiple bilateral transmetatarsal amputations, prior kidney transplant failure, and longstanding hemodialysis presented for urgent skin biopsies due to concern for calciphylaxis (calcific uremic arteriolopathy). She had been on lumasiran for 2 years and her nephrologist believed that her PH1 was well controlled. Serum oxalate levels were not indicated in the patient's chart, but clinical communication indicated serum oxalate levels were substantially reduced since initiation and no new vascular occlusive complications had occurred. As a result, painful thigh ulcerations worsening for 2 months prompted urgent dermatology referral to confirm diagnosis of calciphylaxis and facilitate initiation of sodium thiosulfate chelation therapy. The patient provided limited clinical history and did not initially disclose her PH1 diagnosis, resulting in a clinical focus on her end‐stage renal disease and presumed calciphylaxis.

The patient presented to a rural, outlying non‐dermatology clinic. Due to the level of acute pain and the nephrologist's urgent concern for calciphylaxis, the examination was lesion‐focused. Physical examination showed an ill‐appearing female in acute pain.

On bilateral, anterolateral thighs there were irregular, tender deep ulcerations with indurated violaceous borders (Figure 1). Subcutaneous fat was noted in the ulcer base and borders were not undermined. Two stellate‐like ulcers were present on the right thigh and an oval‐shaped ulcer was on the left thigh. There were prominent, retiform purpura on her thighs and surrounding the ulcerations. In addition, there was a distinct livedo reticularis vascular pattern from mid‐thighs to dorsal feet. Intervening skin was not indurated or tender. Lower legs were somewhat cool, but dorsalis pedis and posterior tibial artery pulses were normal.

**FIGURE 1:**
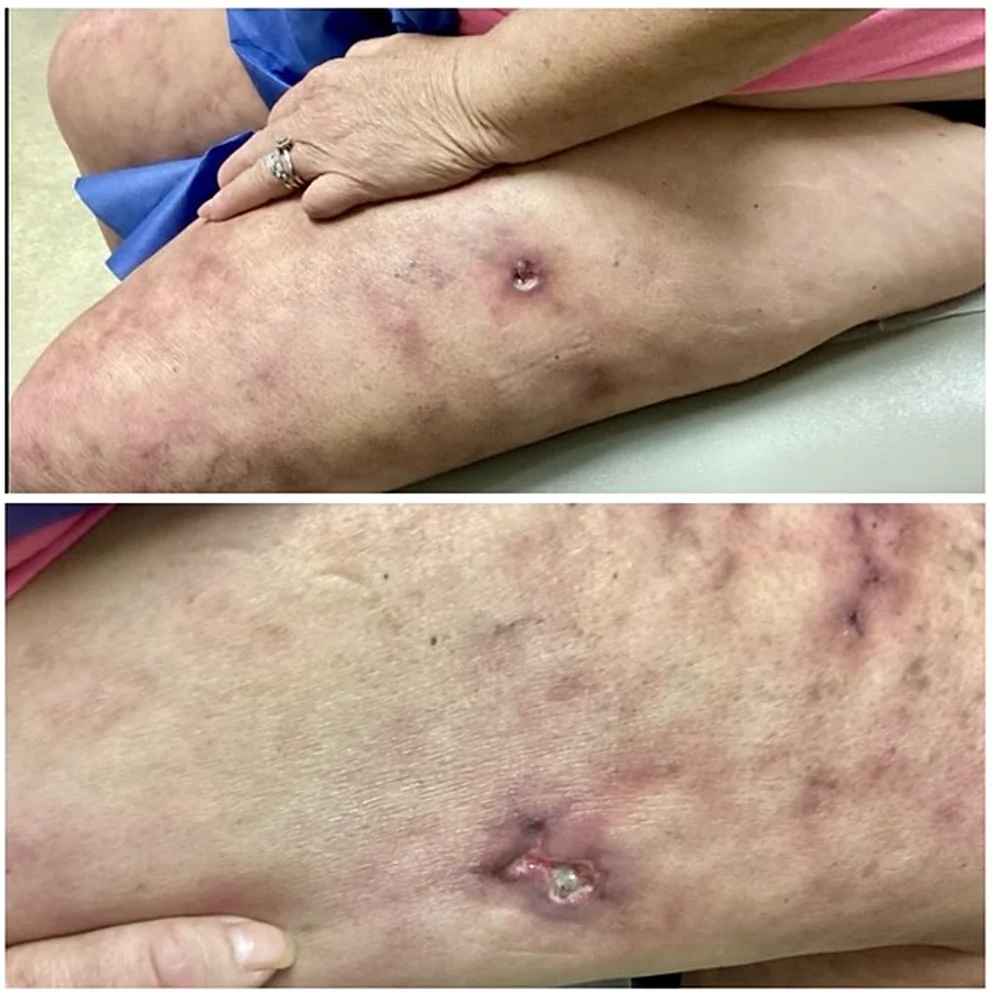
Cutaneous oxalosis, clinical presentation. Left and right thighs with irregular stellate deep ulcerations surrounded by livedo reticularis and foci of retiform purpura at second office visit.

Initially, a tangential shave biopsy of an ulcer edge on the right anterior proximal thigh was performed by an outside, non‐dermatology practitioner. The initial biopsy showed livedoid vasculopathy but lacked sufficient subcutaneous fat to definitively evaluate for deeper vascular calcification. Thus, the patient returned to our dermatology clinic 7 days later for additional deep tissue biopsies.

At this visit, multiple digital amputations were noted, involving distal phalanges of all fingers except thumbs and left fourth finger. The patient was missing all 10 toes; all amputated at metatarsophalangeal joints (Figure 2). Two 6 mm deep punch biopsies were performed on the right posterior‐lateral distal thigh 5–6 mm from the ulcer edge and of noncontiguous retiform purpuric skin.

**FIGURE 2:**
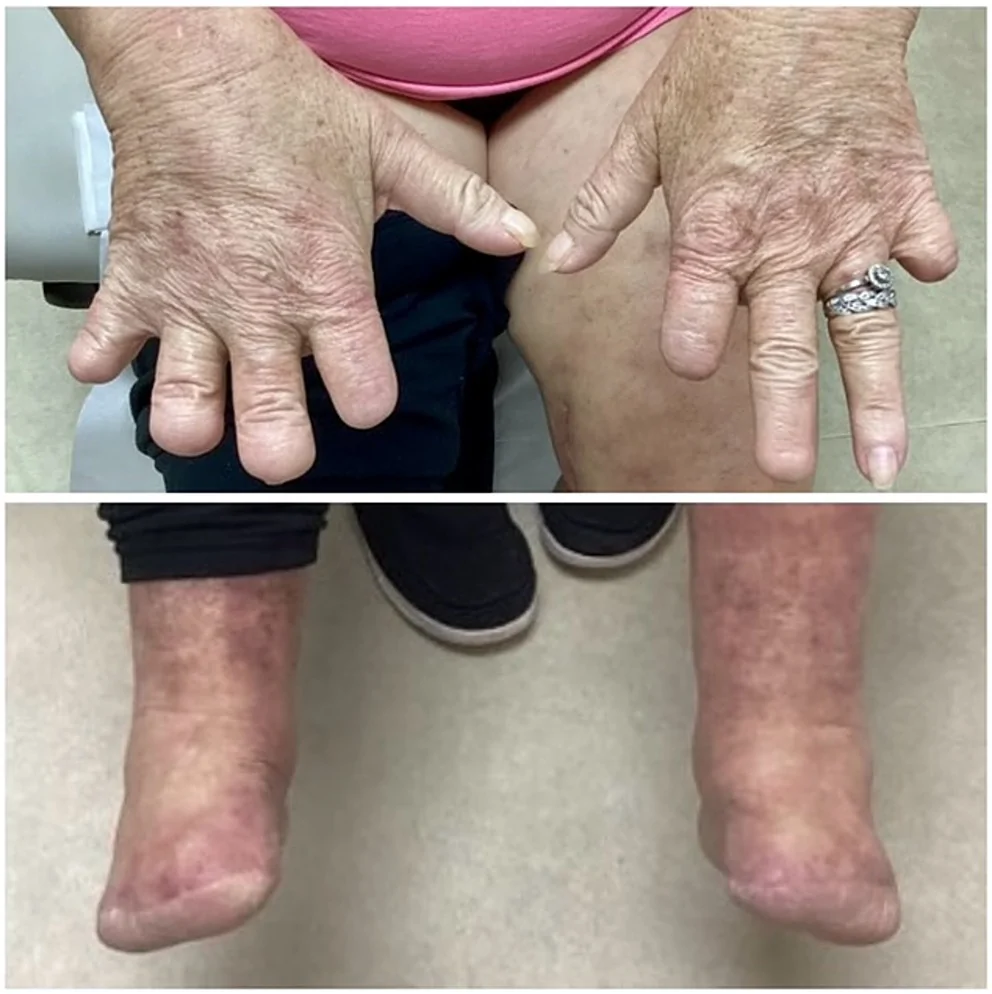
Cutaneous oxalosis, systemic manifestations. Amputations of the distal fingers and all 10 toes.

Microscopic examination of the original deep tangential biopsy showed marked epidermal ulceration with an increased number of small, thick‐walled blood vessels in the upper dermis. Many of these vessels also had fibrin cuffs and some showed fibrin thrombi. In one focus there was a prominent vessel oriented perpendicular to the epidermis showing a long intraluminal hemorrhagic thrombus. Only the dermal‐subcutaneous fat junction and upper subcutaneous fat tissue were present for evaluation. Vasculitis, intimal hyperplasia, mural calcifications, and dermal elastic fiber changes were not identified on deeper sections (Figure 3).

**FIGURE 3:**
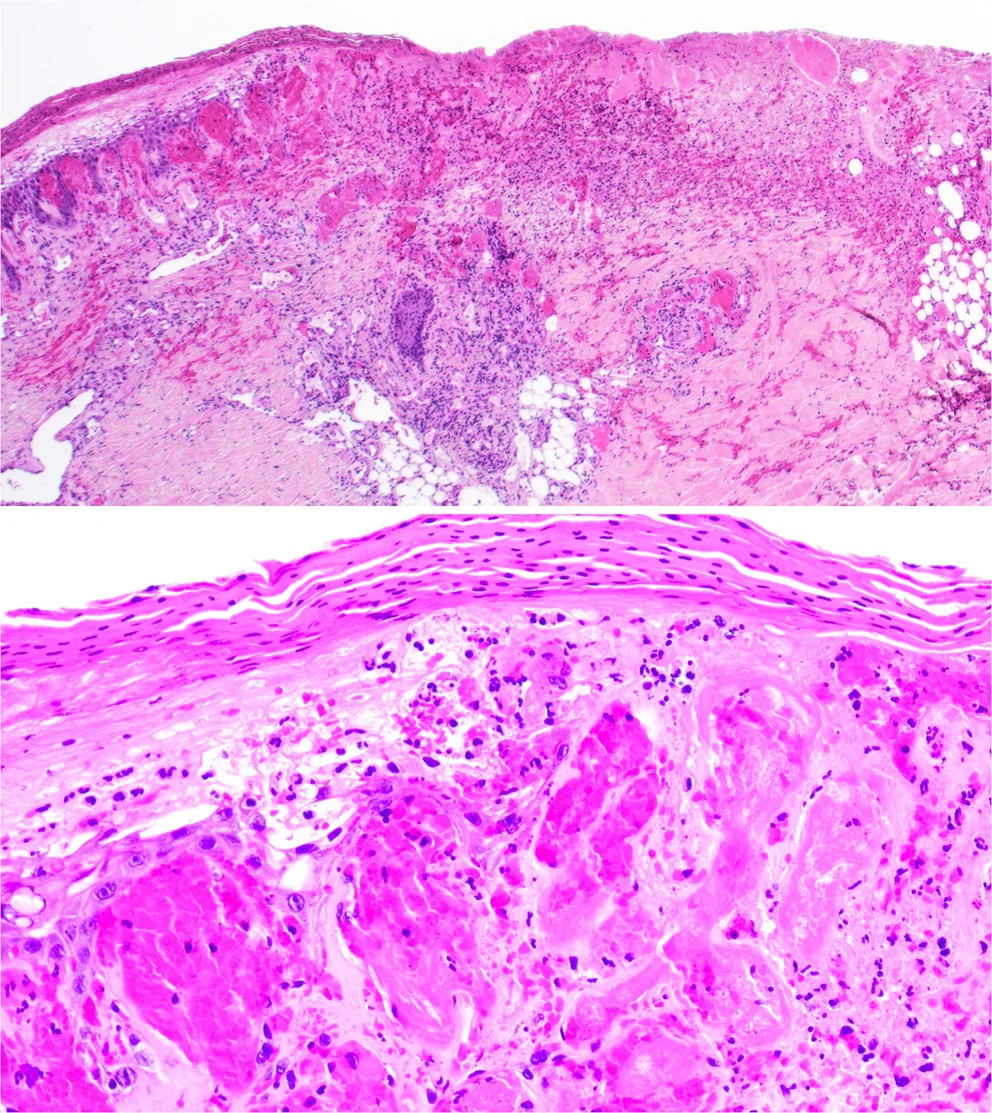
Cutaneous oxalosis. Top: Low‐power photomicrograph showing marked epidermal ulceration, vascular congestion and thrombi (H&E, original magnification 20×). Bottom: Close‐up showing intraluminal thrombi without calcification. Vessel crystalline deposits are absent (H&E, original magnification 100×).

The second punch biopsy from retiform purpura near the right thigh ulcer showed unremarkable epidermis with marked dermal hemorrhage. Step sections showed three medium‐sized vessels at the dermal‐subcutaneous fat junction with lumina occluded by amorphous crystalline material (Figure 4).

**FIGURE 4:**
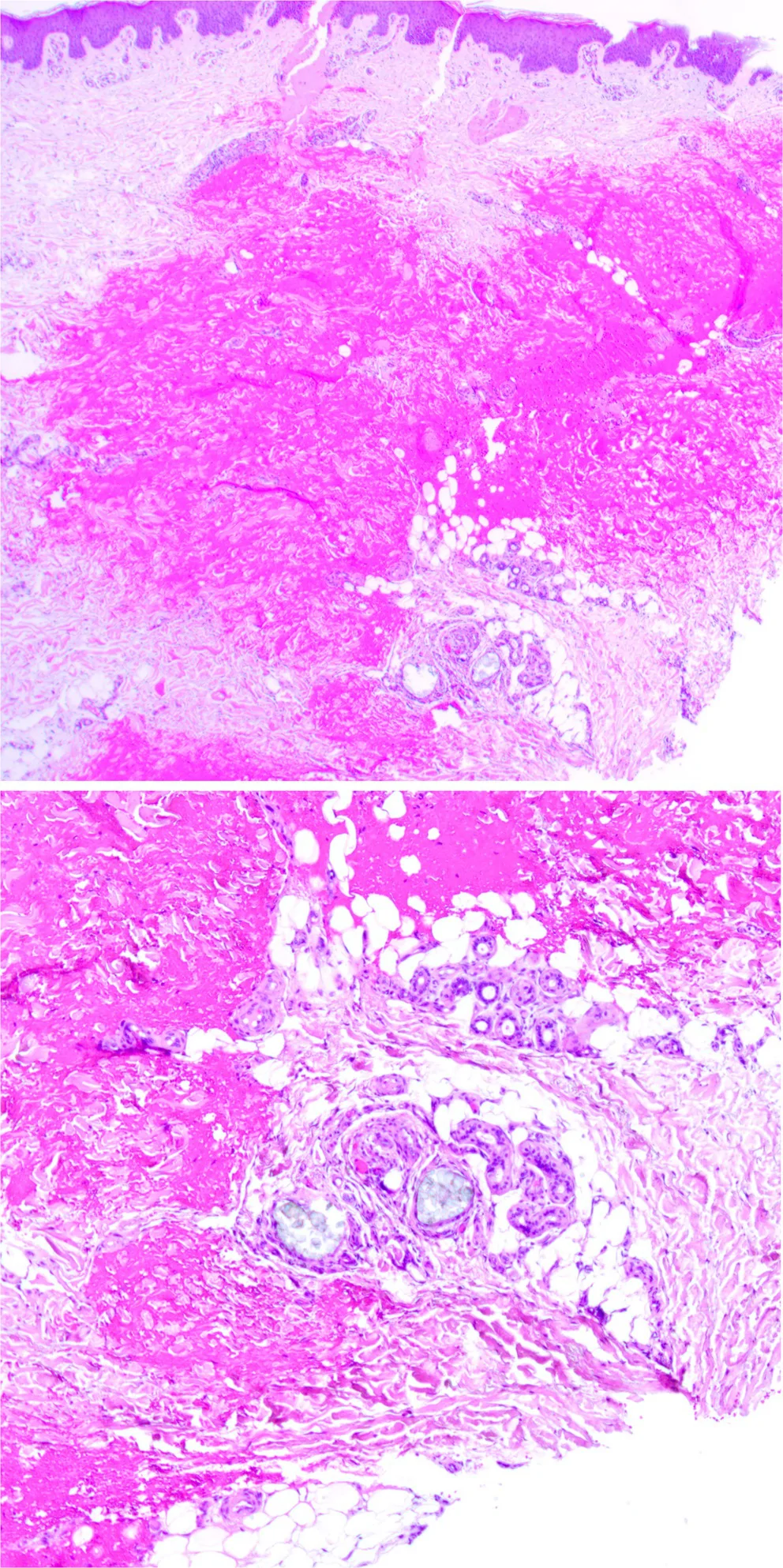
Cutaneous oxalosis, histopathology of deep punch biopsy. Top: Low‐power photomicrograph of second biopsy showing marked dermal hemorrhage (H&E, original magnification 20×). Bottom: Medium‐power photomicrograph of upper subcutaneous fat tissue showing intraluminal crystalline material in vessels without presence of calcification or vessel intimal hyperplasia (H&E, original magnification 40×).

Closer examination showed the intraluminal material to be slightly radially‐arranged rhomboidal, yellowish‐gray crystals. Under polarized light examination, the crystals showed white and multicolored brilliant birefringence. The crystalline deposits were felt to be most consistent with oxalate (Figure 5). To rule out histopathologic mimickers within the vascular lumina, ancillary stains were performed; calcium phosphate was excluded by a negative Von Kossa stain, and uric acid was excluded by a negative Grocott's Methenamine Silver (GMS) stain. Ultimately, the diagnosis was solidified by this distinctive, bright, radial birefringence, which cleanly differentiates calcium oxalate from other crystalline deposits or iatrogenic intravascular hyaluronic acid.

**FIGURE 5:**
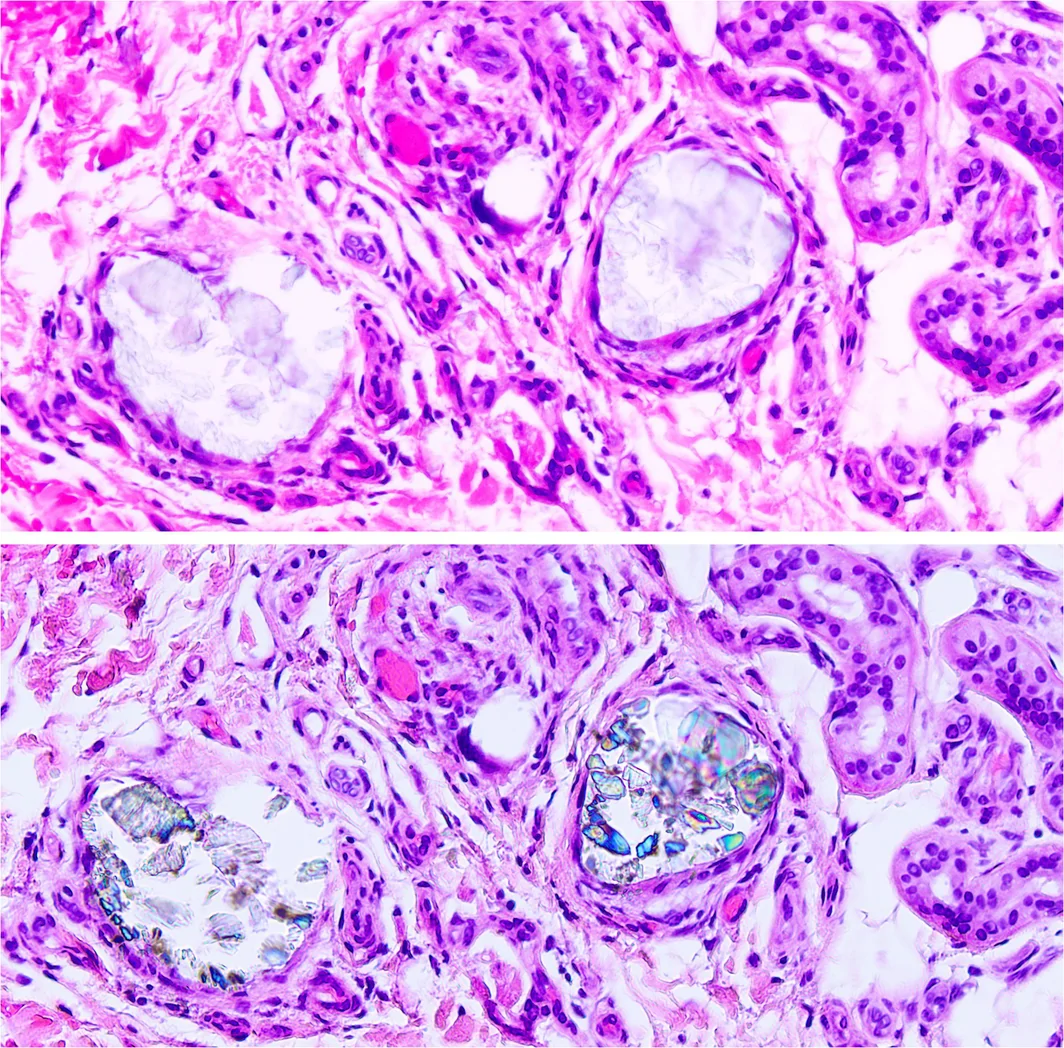
Cutaneous oxalosis, crystalline morphology and polarization. Top: High‐power photomicrograph of intraluminal translucent‐yellowish amorphous‐rhomboidal crystals (H&E, original magnification 200×). Bottom: Identical high‐power field under polarized light examination with strong birefringence characteristic of calcium oxalate crystals (H&E, original magnification 200×).

## 3 Discussion

Primary hyperoxalurias (primary oxalosis) are autosomal recessive disorders of glyoxylate metabolism leading to hepatic overproduction of oxalate [5]. Type 1 primary hyperoxaluria, the most common form, is caused by a mutation in the enzyme, AGT, resulting in uncontrolled production and excess urinary excretion of oxalate [1]. This is in contrast to secondary oxalosis, which typically occurs from an increase in dietary and intestinal absorption (enteric hyperoxaluria) via excess intake of oxalate precursors or disruption in intestinal microflora [6]. Excess vitamin C, a precursor of oxalate, may also result in the precipitation of calcium oxalate [6].

Clinical presentations of primary hyperoxaluria range from urolithiasis to nephrocalcinosis to kidney failure of unknown origin, particularly when echogenic kidneys are identified on ultrasound [5]. If renal damage is progressive and glomerular filtration rate falls below 30–40 mL/min per 1.73 m^2^, renal excretion of calcium oxalate is significantly impaired and systemic deposition of calcium oxalate occurs [6]. Ultimately, many patients require dialysis and kidney or liver transplantation in childhood due to insoluble calcium oxalate crystal deposition and progressive damage to internal organs [5].

As a result, patients with ESRD are at increased risk of developing secondary oxalosis. Skin presentations include ischemic cutaneous ulcers, livedo reticularis, subcutaneous nodules, and calciphylaxis‐like cutaneous necrosis [5]. Calciphylaxis is a complication of ESRD that occurs when calcium deposits in dermal and adipose microvasculature [7]. This results in livedo reticularis which can progress to retiform purpura and skin necrosis, predisposing patients to skin infections with possible progression to sepsis and death [7].

In one reported case, a patient with lupus nephritis complicated by ESRD on peritoneal dialysis presented with hyperpigmented macules and patches overlying firm papules, nodules, and plaques on bilateral upper and lower extremities that were painful with pressure [8]. Due to the history of ESRD with peritoneal dialysis and the presence of cutaneous papules and nodules, calcinosis cutis was strongly suspected, but biopsy later confirmed cutaneous oxalosis likely secondary to her ESRD [8]. Although this represents a single case, it underscores the importance of maintaining a broad differential diagnosis when evaluating painful skin ulcerations with retiform purpura in the setting of ESRD, as early diagnosis can help tailor appropriate management strategies [8].

Current treatment options for oxalurias include traditional options such as hyperhydration to alleviate stone burden, crystallization inhibitors to reduce calcium oxalate precipitation in urine, and pyridoxine supplementation which acts as a cofactor for AGT enzyme to help enhance enzymatic activity (specifically for PH1) [6, 9]. More intensive therapies include dialysis to remove overproduced oxalate and maintain levels as low as possible and eventual transplantation of liver and/or kidney if organ damage is extensive [9]. Lumasiran, a synthetic double‐stranded RNA interference molecule, has been approved for patients of all ages and at all stages of kidney function with PH1. It has been shown effective in reducing hepatic oxalate production, lowering oxalate levels in urine and/or plasma, and helping preserve kidney function in both pediatric and adult patients, irrespective of whether they were receiving concurrent dialysis [10]. Overall efficacy in clinical trials showed that while lumasiran significantly lowers systemic disease burden, only 52% of patients achieved completely normal urinary oxalate levels at 6 months and 16% failed to even reach near normal parameters [11]. This underscores that none of these therapies are curative; rather, they aim to slow disease progression and increase lifespan. As a result, significant morbidity and mortality can occur due to ESRD, especially if oxaluria is not treated early in disease course [6].

Calciphylaxis is characterized by microvascular calcification and thrombosis that leads to tissue ischemia, necrosis, and painful lesions. While rare, it is more common in patients with ESRD on dialysis [12]. Because cutaneous manifestations of calciphylaxis can resemble those of oxalosis, biopsy is typically required for diagnosis. As illustrated by our case, microscopic findings of oxalate crystals can be subtle or focal, particularly in superficial samples. Therefore, obtaining multiple biopsies and deeper sections may be necessary to establish an accurate diagnosis. From a histopathologic standpoint, distinguishing intravascular oxalosis from common mimickers necessitates meticulous microscopic evaluation. Von Kossa or Alizarin Red should be utilized for suspected calciphylaxis, which will highlight basophilic, amorphous calcium deposition within vessel walls [12]. Other crystalline or amorphous mimics, like intravascular uric acid deposition and hyaluronic acid fillers, can also be distinguished from oxalate deposits. Uric acid deposits will demonstrate needle‐shaped crystals with GMS stain while hyaluronic acid appears as non‐crystalline amorphous gel on routine H&E [8]. However, histopathologic diagnosis of calcium oxalate must include polarized light examination in order to visualize the strong, multicolored birefringence [2, 3]. Because these crystalline structures can be focal and located only in the deeper vascular structures, executing multiple, deep step sections to the subcutaneous layer is a key diagnostic step to avoid false‐negative results on superficial tissue biopsy.

Although our patient had a known diagnosis of primary hyperoxaluria, her disease was considered well‐controlled with lumasiran. From a histopathologic perspective, this underscores a critical diagnostic pitfall: cutaneous and systemic oxalate deposition with focal crystal formation may still manifest in microvasculature despite medical management with promising small interfering RNA therapy and perceived stable metabolic control. Dermatologic evaluation was initiated due to a strong clinical suspicion for calciphylaxis, and initial limited history, which did not mention PH1, contributed to a delay in diagnosis. This case highlights how primary hyperoxaluria can affect multiple organ systems and how its cutaneous manifestations may resemble other complications of ESRD, such as calciphylaxis. Oxalosis, whether primary or secondary, should remain part of the differential when evaluating ulcers and retiform purpura, even in patients with ESRD on dialysis where calciphylaxis is strongly suspected.

## Conflicts of Interest

The authors declare no conflicts of interest.

## Data Availability

Data sharing not applicable to this article as no datasets were generated or analysed during the current study.
